# Anomalous orbital structure in two-dimensional titanium dichalcogenides

**DOI:** 10.1038/s41598-018-37248-5

**Published:** 2019-02-13

**Authors:** Banabir Pal, Yanwei Cao, Xiaoran Liu, Fangdi Wen, M. Kareev, A. T. N’Diaye, P. Shafer, E. Arenholz, J. Chakhalian

**Affiliations:** 1 Department of Physics and Astronomy, Rutgers University, Piscataway, New Jersey 08854 USA; 2 Ningbo Institute of Materials Technology and Engineering, Chinese Academy of Sciences, Ningbo, Zhejiang 315201 China; 3 Advanced Light Source, Lawrence Berkley National Laboratory, Berkeley, California 94720 USA

## Abstract

Generally, lattice distortions play a key role in determining the electronic ground states of materials. Although it is well known that trigonal distortions are generic to most two dimensional transition metal dichalcogenides, the impact of this structural distortion on the electronic structure and topological properties has not been understood conclusively. Here, by using a combination of polarization dependent X-ray absorption spectroscopy (XAS), X-ray photoelectron spectroscopy (XPS) and atomic multiplet cluster calculations, we have investigated the electronic structure of titanium dichalcogenides TiX_2_ (X = S, Se, Te), where the magnitude of the trigonal distortion increase monotonically from S to Se and Te. Our results reveal the presence of an anomalously large crystal field splitting. This unusual kind of crystal field splitting is likely responsible for the unconventional electronic structure of TiX_2_ compounds and ultimately controls the degree of the electronic phase protection. Our findings also indicate the drawback of the distorted crystal field picture in explaining the observed electronic ground state and emphasize the key importance of trigonal symmetry, metal-ligand hybridization and electron-electron correlations in defining the electronic structures at the Fermi energy.

## Introduction

The realization of numerous exotic electronic phases of graphene^1–3^ and the relentless tendency to miniaturization of silicon-based electronics^4^ have ignited exhaustive research in a wide range of two dimensional (2D) layered materials. As a result, 2D transition metal dichalcogenides have emerged as the promising platform with intriguing topological and electronic ground states and high potential for applicability in the field of microelectronics^5,6^, nanophotonics^7,8^, optoelectronics^9,10^ and photovoltaics^11–13^ to name a few.

A generic feature of this dichalcogenide family is the presence of structural distortions which play a critical role in defining the electronic ground state and invariably the topological properties of these systems. Specifically, the lattice deformations can strongly alter the interatomic interaction strength and thereby are responsible for various novel electronic phases including charge density wave in VSe_2_^14,15^, NbSe_2_^16^ and TaSe_2_^17,18^, superconductivity in FeSe_1−*x*_Te_*x*_^19^, insulating ground states in ReSe_2_^20^, Weyl semi-metallic phase in MoTe_2_^21^ and many more. On the other hand, recent results on topological phases (TPs) have demonstrated a remarkable degree of protection against perturbations as long as the key symmetries remain intact^22^. Experimentally, surprisingly very little is know about the TP stability subjected to lattice distortions associated with trigonal symmetry as trigonal distortions could be used to either enter a topological phase, or exit a topological phase with a trigonal distortion^22–25^. Naturally, a way to microscopically probe and understand the effect of lattice deformations, induced covalency, and electron-electron correlations along with their impact on the electronic structure are essential for deterministic control of the rich physical phases of these TMDs compounds.

Recently, titanium dichalcogenides TiX_2_ (here X = S, Se and Te) have attracted significant attention of the community as the exemplary TMD systems^26–30^. While the usual crystallographic form of TiX_2_ is the layered CdI_2_ type^31^, these systems also possess a distinct trigonal distortion from an ideal octahedral crystal environment^31,32^. Previously, detailed structural investigation established that the magnitude of the distortion varies monotonically from nearly octahedral in TiS_2_^31^ to highly distorted in TiTe_2_^32^. In addition, large body of work on transition metal compounds suggest that these generic trigonal distortions may have strong influence in modifying the energy level and electronic structure of the chalcogenides. The problem is vividly illustrated by the case of TiSe_2_ which undergoes the transition into a chiral charge density wave (CDW) state^33,34^ and further into a conventional CDW resulting in dramatic renormalization of electronic and structural properties. The fundamental challenge here is to separate the many-body effects associated with excitonic condensate^35–37^ from the Jahn-Teller like instability from the strong-electron phonon coupling^38,39^. In addition, recent extensive theoretical work based on LDA + U^40^ along with the detailed ARPES^41^ study hinted on the importance of lattice distortions coupled with strong electron-electron correlations to explain the ground state properties of TiSe_2_. Based on those findings and given the obvious importance of these the whole TiX_2_ family, there have been several experimental and theoretical investigations to understand the impact of trigonal distortion on the electronic structure in these systems^42–44^. However, in absence of systematic spectroscopic investigations corroborated with theoretical calculations the consequence of the trigonal distortion on the electronic structure of the TiX_2_ systems has not convincingly been reported thus far. Here, we have addressed this issue by investigating the whole family of TiX_2_ single crystals by means of polarization dependent X-ray absorption spectroscopy in conjunction with the multiplet cluster calculations. Our results unambiguously demonstrate the failure of the standard ionic configuration and distorted crystal field picture in predicting electronic ground states and thereby reveal the key importance of metal-ligand hybridization between titanium and chalcogen ions and electronic correlations in defining their electronic properties.

## Results and Discussion

**Figure 1:**
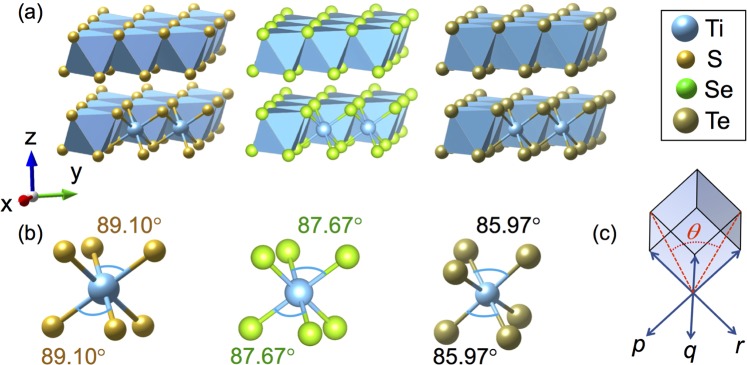
(**a**) Schematic crystal structure of the TiS_2_, TiSe_2_, and TiTe_2_ compounds showing nearly octahedral environment of each TiX_2_ unit. (**b**) Distortion of the ideal octahedral environment across the chalcogenide series. It is to be noted that the distortion does not change the bond length but alters the bond angle. (**c**) Schematic representation of the trigonal distortions.

As shown in Fig. 1(a), all members of the TiX_2_ family crystallize into trigonal CdI_2_ type layered structure with space group *P*_3*m*1_^31^. The crystal structure consists of repeated tri-layers (Fig. 1(a)) along crystallographic *z* direction; each tri-layer in these systems contains a titanium layer sandwiched between two layers of chalcogenides. Although the interactions between titanium and chalcogenides are strong within a tri-layer, the chalcogen bonding between two tri-layers is weak and dominated by the weak van der Waals type interaction. Each tri-layer further experiences an elongated trigonal distortion where all six Ti-X bond length remains constant but X-Ti-X bond angle deviates from an ideal 90° in such a manner that the crystallographic lattice parameter along *z* direction increases in length. As illustrated in Fig. 1(b) the strength of elongated trigonal distortion increases monotonically from TiS_2_ to TiTe_2_ with three different X-Ti-X bond angles. The magnitude of such elongated trigonal distortion can be estimated from *c*/*a* ratio where *c* and *a* represent the corresponding unit cell parameter along *z* and *x* direction, respectively; For an ideal octahedral environment, the *c*/*a* ratio is close to 1.633 whereas the *c*/*a* ratio for TiS_2_, TiSe_2_ and TiTe_2_ are found to be 1.726, 1.732 and 1.808, respectively. This type of structural distortion can also be alternatively explained in terms of the distortion angle Θ between the diagonals of the *pq*, *qr*, *rp* plane as schematically shown in Fig. 1(c). For a regular octahedron Θ = 60° and becomes lesser and greater than 60° for elongated and compressive trigonal distortions, respectively.

**Figure 2:**
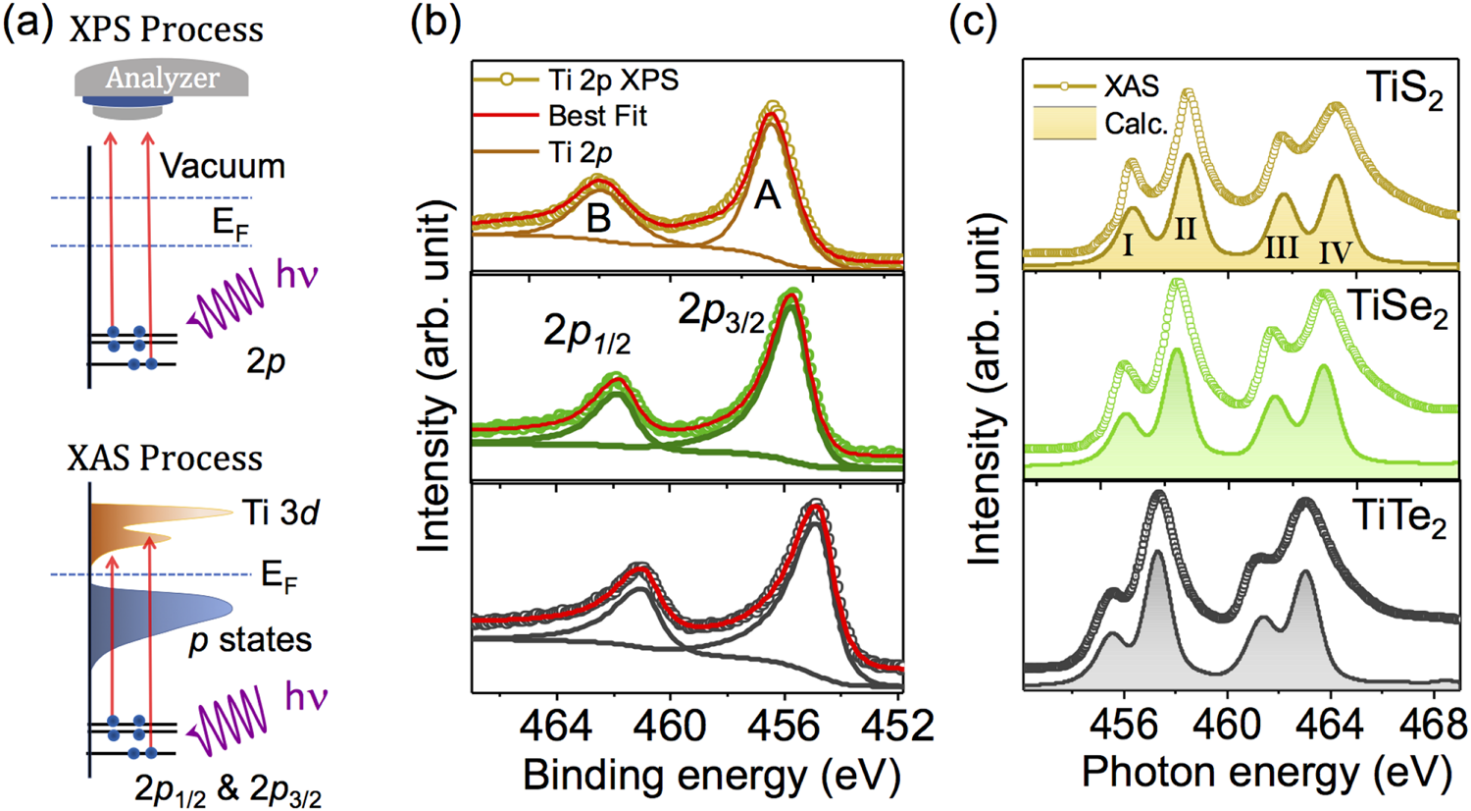
(**a**) Schematic representations of the X-ray photoelectron (top) and X-ray absorption (bottom) spectroscopy process. (**b**) X-ray photoelectron spectra of Ti 2*p* core level. Each spectrum was decomposed with Lorentzian function convoluted with Gaussian functions. (**c**) Experimental XAS spectra (open circle) of three different TiX_2_ systems were compared with atomic multiplet calculations (shaded area).

Chemical quality, and the absence of chalcogen vacancies critical for most dichalcogenides are verified by X-ray photo electron spectroscopy (XPS) measurements carried out on freshly cleaved TiX_2_ single crystals to rule out the presence of any anion vacancies. The basic process associated with the XPS technique is shown schematically in Fig. 2(a). Generally, the chemical shift associated with each core level provides important information about the charge state of the different elements of the system under investigation. Figure 2(b) displays typical Ti 2*p* core level spectra for three different TiX_2_ systems (Core level XPS spectra of Chalcogenides are shown in Supporting Information). Each Ti 2*p* core-level spectrum shown in Fig. 2(b) is composed of two intense spin-orbit split doublet (marked as A and B) with a spin-orbit splitting strength close to 5.8 eV. Feature A appearing at a binding energy range between 454 eV to 459 eV represents Ti 2*p*_3/2_ like states whereas feature B arises mainly from Ti 2*p*_1/2_ like states. Interestingly, a systematic shift in peak position was found from higher to lower binding energy in Ti 2*p* core level spectra from TiS_2_ to TiTe_2_, respectively. These shifts in the binding energy were previously attributed to the reduction in ionic contribution in Ti-X chemical bond formations^45^. In this work, each spectrum was decomposed using a Gaussian-Lorentz line profile and a Shirley type background function and can be accounted within a single Gaussian-Lorentz line profile. The absence of the multiple peak structure in Ti 2*p* core level spectra clearly implies the presence single valence Ti, and rules out any signature of anion vacancies in our TiX_2_ samples.

**Figure 3:**
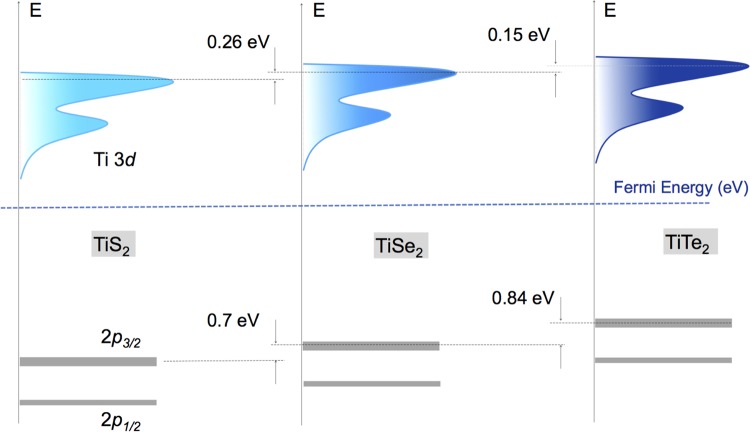
(**a**) Band alignment of 2*p* and 3*d* states in TiS_2_, TiSe_2_ and TiTe_2_ as obtained from X-ray photoelectron and X-ray absorption spectroscopy.

To investigate the evolutions in electronic structure across the TiX_2_ series, XAS measurements were carried out on Ti L_2,3_ edge at beamline 4.0.2 of the Advanced Light Source, at Lawrence Berkeley National Laboratory. In a typical Ti L_3,2_ XAS process (see bottom of Fig. 2(a)), electrons are excited from Ti 2*p* core level (from 2*p*_3/2_ and 2*p*_1/2_) to the Ti 3*d* conduction band; each L_3,2_ XAS spectrum splits into two edges (L_2_ and L_3_) due to the spin-orbit coupling of Ti 2*p* states. Experimentally obtained Ti L_2,3_ XAS spectra for three different TiX_2_ systems are shown in Fig. 2(c) as open circles. As seen both L_3_ and L_2_ peaks of each spectrum exhibit a double hump feature arising primarily from nearly octahedral crystal field effects which splits the fivefold degenerate 3*d* orbitals of Ti into doubly degenerate *e*_*g*_ and triply degenerate *t*_2*g*_ orbitals. For a quantitative understanding, theoretical atomic multiplet cluster calculations were performed within a TiX_6_ cluster with O_*h*_ point group symmetry and Ti^4 +^ ionic configuration^46^. The obtained calculated spectra are shown as shaded line in Fig. 2(c) confirm that only one types of charge state Ti^4 +^ is present in our samples. In good agreement with the XPS spectra, we observed a gradual shift in the XAS spectra from TiS_2_ to TiTe_2_. This monotonic spectral shift suggests a systematic decrease in (*U*_*dd*_ − *U*_*pd*_) from TiS_2_ to TiTe_2_ where *U*_*dd*_ symbolizes the onsite coulomb interaction strength and *U*_*pd*_ defines the core hole interaction potential. This result indicates that in case of Te, the strongest interatomic *p* − *d* interaction strength arises from the markedly increased metal-ligand hybridization. The summary of the relative energy position of the occupied 2*p* states and unoccupied 3*d* states of titanium obtained from XPS and XAS measurements is given schematically in Fig. 3.

**Figure 4:**
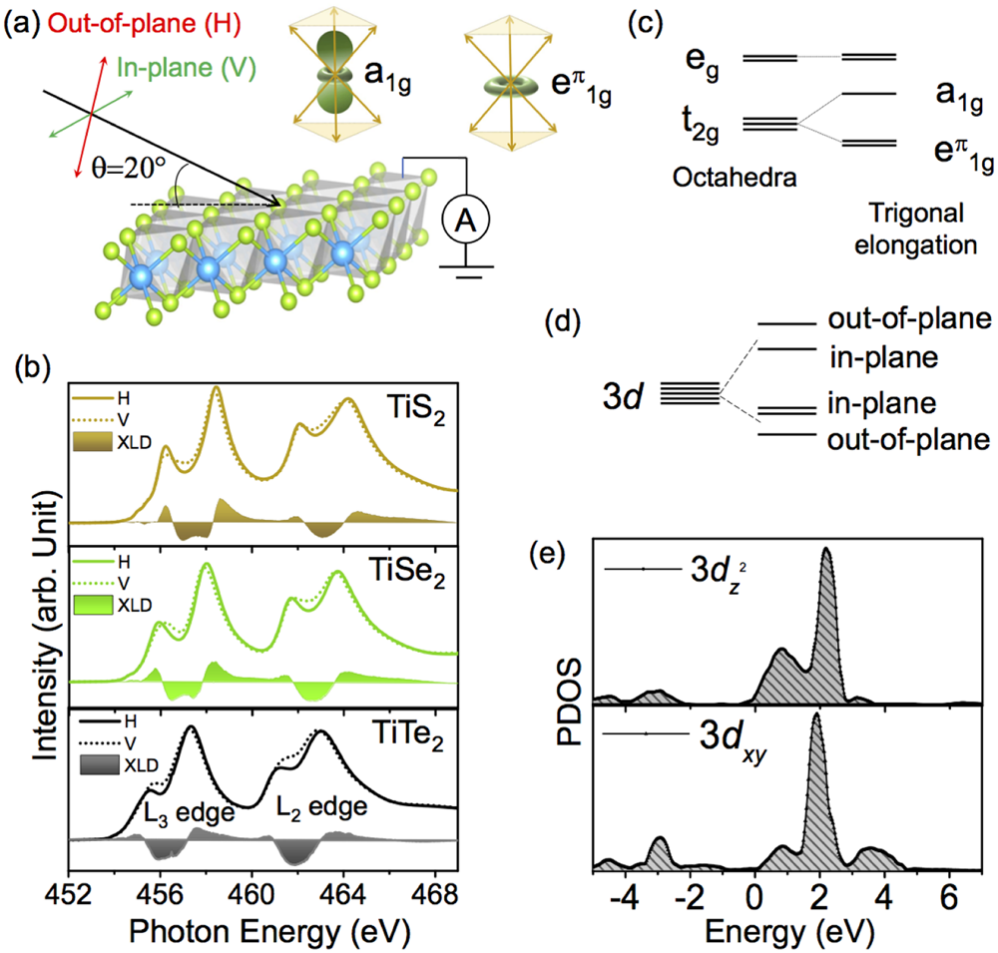
(**a**) Experimental setup of the in-plane and out-of-plane polarization dependent XAS measurements. (**b**) Ti L_3,2_ edge XAS/XLD spectra of three different TiX_2_ compounds at room temperature. (**c**) Conventional theoretical model of an elongated trigonal distortion with *t*_2*g*_ sub-band splitting. (**d**) Experimentally observed Ti 3*d* sub-band splitting in TiX_2_. (**e**) PDOS of 3${d}_{{z}^{2}}$ and 3*d*_*xy*_/3${d}_{{x}^{2}-{y}^{2}}$ obtained from previous theoretical calculations^49^ on TiS_2_ system where *x*, *y*, *z* represent global co-ordinates axis as shown schematically in Fig. 1(a).

Despite high XAS sensitivity to charge state and local symmetry, it is still very challenging to capture the effect of trigonal distortions from non-polarized XAS measurement. To further explore the impact of trigonal distortion, detailed polarization dependent XAS measurements were carried out. Polarization dependent XAS process can probe local orbital character depending on their relative orientation with respect to crystallographic axes; for example it can be sensitive to the sub-band splitting of *t*_2*g*_ states which may emerge from local lattice distortions. The process associated with the polarization dependent XAS (in-plane vs. out-of-plane) are schematically shown in Fig. 4(a). Since our samples are aligned along their natural [111] direction (z axis in Fig. 1(a) is along [111] direction), it is expected that out-of-plane polarization will be sensitive to the orbitals oriented along [111] direction whereas the in-plane polarization will probe those states which are oriented perpendicular to the [111] directions. Both out-of-plane (H) and in-plane (V) polarization dependent spectra are shown in Fig. 4(b). As immediately seen, a clear difference between the in- and out-of-plane XAS spectra is present which in turn implies the presence of distinct *d*-orbital anisotropy. The corresponding XLD signal or the difference between the in- and out-of-plane XAS spectrum is shown as the shaded area in Fig. 4(b) for all three samples.

To make a connection between the XLD results and orbital occupation we discuss electronic structure of the TiX_2_ compounds. In the purely ionic limit, the electronic structure of these systems comprises of fully occupied *p* states of chalcogen atom (S or Se or Te) forming valence bands and unoccupied conduction bands with the predominant Ti 3*d* character. Due to the effect of octahedral crystal field, five-fold degenerate 3*d* orbitals of Ti split into a doubly degenerate higher energy *e*_*g*_ and a triply degenerate lower energy *t*_2*g*_ states^32^. In addition, as shown schematically in Fig. 4(c) due to elongated trigonal distortion^47^ *t*_2*g*_ states further split into a higher energy singlet *a*_1*g*_ and lower energy doublet ${e}_{g}^{\pi }$ states. The wave function of *a*_1*g*_ and ${e}_{g}^{\pi }$ states is a linear combination of *d*_*xy*_, *d*_*yz*_ and *d*_*xz*_ orbitals and can be expressed as as $|{a}_{1g}\rangle =\frac{1}{\sqrt{3}}(|xy\rangle +|yz\rangle +|xz\rangle )$ and $|{e}_{g\pm }^{\pi }\rangle =\pm \,\frac{1}{\sqrt{3}}(|xy\rangle +{e}^{\mp 2i\pi /3}|yz\rangle +{e}^{\pm 2i\pi /3}|xz\rangle )$^48^. The description of the mixed *a*_1*g*_ and ${e}_{g}^{\pi }$ states becomes simplified when one assumes *z* axis along the [111] direction as shown in Fig. 1(a). The wave function of the *a*_1*g*_ state has a similar shape along [111] direction as the ${d}_{{z}^{2}}$ orbital along [001]; ${e}_{g}^{\pi }$ states are perpendicular to the [111] directions and oriented in *xy* plane (see the shape of the *a*_1*g*_ and ${e}_{g}^{\pi }$ orbital Fig. 4(a)).

Based on this picture, one would naively expect that in-plane polarization will largely probe ${e}_{g}^{\pi }$ states which are at lower energy compared to the higher energy *a*_1*g*_ state that are more sensitive to out-of-plane polarization. Contrary to this expectation, the experimental spectra for all three systems are in complete variation with the conventional picture. More specifically, in the case of *t*_2*g*_ states labeled by feature I and III in Fig. 2(c), the out-of-plane polarization (shown by solid line in Fig. 4(b)) exhibits a lower energy peak position compared to the in-plane polarization (shown by dotted line). This implies the stabilization of *a*_1*g*_ as the lowest occupied orbital contrary to the expected ${e}_{g}^{\pi }$ state. This is completely opposite to what one would infer from the standard crystals field splitting arguments. The crystal field inversion picture is schematically shown in Fig. 4(d) (Quantitative Sub-band splitting of each TiX_2_ compound has been shown schematically in Supporting Information). These findings clearly suggest that purely ionic picture which is normally very efficient in capturing the excitation spectra of Ti^4 +^ derived states in oxide systems is inadequate in explaining the origin of XLD signal in dichalcogenides. Several factors might be responsible for such discrepancy in the description of the XAS spectra. Because of the markedly enlarged *p*-orbitals in S, Se and especially Te, unlike oxides, covalency may play a dominant role in transition metal dichalcogenides. The synergistic effect of high metal-ligand hybridization and significant ligand field can strongly affect the local electronic description of these systems.

Adding more to the surprising finding, the crystal field splitting gap (10 *Dq*) between *t*_2*g*_ and *e*_*g*_ is found to be strongly dependent on the direction of polarization of the incident light. In particular, out-of-plane polarization (solid line in Fig. 4(b)) shows a higher crystal field gap compared to in-plane polarizations (dotted line in Fig. 4(b)). This is unexpected since for the trigonal distortion, the *e*_*g*_ states should not experience any sub-band splitting. However, the energy position of the *e*_*g*_ state is found to depend strongly on the types of polarization. All these discrepancies clearly establish that conventional crystal field picture fails to explain the observed features in these system contrary to the Ti^4+^ based oxide materials.

To resolve this inconsistency, the XAS spectra were compared with calculated projected partial density of states (PDOS) of 3${d}_{{z}^{2}}$ and 3*d*_*xy*_ states of TiS_2_^45,49–51^ as shown in Fig. 4(e). Interestingly, the calculated PDOS of 3${d}_{{z}^{2}}$ of titanium exhibit a double hump structure analogous to the experiments when the systems were probed with out-of-plane polarization and PDOS of the 3*d*_*xy*_/3${d}_{{x}^{2}-{y}^{2}}$ states (these two states are degenerate) also shows a double hump feature similar to in-plane polarization. Moreover, the energy gap between the doublet features is greater in the case of 3${d}_{{z}^{2}}$ PDOS as compared to 3*d*_*xy*_, and 3${d}_{{x}^{2}-{y}^{2}}$ PDOS which is remarkably similar to the experimentally observed trend. The calculated PDOS is qualitatively consistent with experimental observations and explains the appearance of the all features and their energy position as observed from experiments. The key feature of the theoretical calculation is the inclusion of strong metal-chalcogen hybridization which is responsible for the observed crystal field inversion and allows to capture the details of electronic structure within the band-like description. This result implies that indeed covalency and metal-ligand hybridization are critical for the electronic properties of the TiX_2_ family of materials.

In conclusion, detailed polarization dependent XAS measurements were carried out to probe and microscopically understand the effect of trigonal lattice distortion on the electronic structure of the TiX_2_ family. All systems were characterized to rule out the presence of anion vacancies in these compounds. XLD spectra demonstrate the failure of conventional crystal field arguments in explaining the observed experimental features implying the crucial importance of covalency/metal-ligand hybridization in defining the electronic structure. Orbital projected DOS successfully reproduced the spectral features observed in our experiments. The excellent agreement between theory and the XAS spectra suggests the importance of the band-like description including trigonal lattice deformations, electron-electron correlations and metal-ligand covalency to elucidate the electronic structure of transition metal dichalcogenides.

## Methods

### XPS measurements

Ti 2*p*, S 2*p*, Se 3*d* and Te 3*d* core level XPS measurements were carried out in a lab-based Thermo Scientific X-ray Photoelectron Spectrometer furnished with a monochromatic Al *K*_*α*_ photon source and a hemi-spherical analyser with total energy resolution close to 0.45 eV. The base pressure of the main chamber was below 2 × 10^−8^ mbar during XPS measurements. All three TiX_2_ compounds were mechanically exfoliated just before XPS measurements to avoid any surface contamination. The bulk sensitivity of the measurements was increased by collecting the ejected photoelectrons in a surface normal geometry. Photon energy of the source was calibrated using C 1 *s* core level spectra with a characteristic peak at around 284.6 eV binding energy. Each core level spectrum was decomposed in a casaXPS software using Gaussian-Lorentz type line profile.

### XAS /XLD measurements

XAS/XLD measurements on three TiX_2_ compounds were carried out at Ti L_3,2_ edge at beamline 4.0.2 of the Advanced Light Source (ALS), at Lawrence Berkeley National Laboratory, USA. To avoid surface contamination, each single crystal was exfoliated mechanically in nitrogen atmosphere just before carrying out XAS measurements. No further surface treatments were performed as like vacuum heating or sputtering to clean the surface as these techniques could produce chalcogen vacancy in our systems. Total electron yield (TEY) detection technique were used during the XAS/XLD measurements.

## Supplementary information

Supporting information

## References

[1] Castro Neto AH, Guinea F, Peres NMR, Novoselov KS, Geim AK (2009). The electronic properties of graphene. Rev. Mod. Phys..

[2] Allen MJ, Tung VC, Kaner RB (2010). Honeycomb Carbon: A Review of Graphene. Chem. Rev..

[3] Kumar S, Chatterjee K (2016). Comprehensive Review on the Use of Graphene-Based Substrates for Regenerative Medicine and Biomedical Devices. ACS Appl. Mater. Interfaces.

[4] Frazier AB, Warrington RO, Friedrich C (1995). The Miniaturization Technologies: Past, Present, and Future. IEEE Trans. Ind. Appl..

[5] Gong C (2013). Band alignment of two-dimensional transition metal dichalcogenides: Application in tunnel field effect transistors. Appl. Phys. Lett..

[6] Podzorov V, Gershenson ME, Kloc C, Zeis R, Bucher E (2004). High-mobility field-effect transistors based on transition metal dichalcogenides. Appl. Phys. Lett..

[7] Xia F, Wang H, Xiao D, Dubey M, Ramasubramaniam A (2014). Two-dimensional material nanophotonics. Nat. Photonics.

[8] Mak KF, Shan J (2016). Photonics and optoelectronics of 2D semiconductor transition metal dichalcogenides. Nat. Photonics.

[9] Wang QH, Zadeh KK, Kis A, Coleman JN, Strano MS (2012). Electronics and optoelectronics of two-dimensional transition metal dichalcogenides. Nat. Nanotechnol..

[10] Baugher BWH, Churchill HOH, Yang Y, Jarillo-Herrero P (2014). Optoelectronic devices based on electrically tunable p-n diodes in a monolayer dichalcogenide. Nat. Nanotechnol.

[11] Bernardi M, Palummo M, Grossman JC (2013). Extraordinary Sunlight Absorption and One Nanometer Thick Photovoltaics Using Two-Dimensional Monolayer Materials. Nano Lett..

[12] Britnell L (2013). Strong Light-Matter Interactions in Heterostructures of Atomically Thin Films. Science.

[13] Wi S (2014). Enhancement of Photovoltaic Response in Multilayer MoS_2_ Induced by Plasma Doping. ACS Nano.

[14] Tsutsumi K (1982). X-ray-diffraction study of the periodic lattice distortion associated with a charge-density wave in 1T-VSe_2_. Phys. Rev. B.

[15] Woolley AM, Wexler G (1977). Band structures and Fermi surfaces for 1T-TaS_2_, 1T-TaSe_2_ and 1T-VSe_2_. J. Phys. C.

[16] Xi X (2015). Strongly enhanced charge-density-wave order in monolayer NbSe_2_. Nat. Nanotechnol.

[17] Zhu P (2013). Dynamic separation of electron excitation and lattice heating during the photoinduced melting of the periodic lattice distortion in 2H-TaSe_2_. Appl. Phys. Lett..

[18] Dai J (2014). Microscopic evidence for strong periodic lattice distortion in two-dimensional charge-density wave systems. Phys. Rev. B.

[19] Ingle KE (2015). Importance of structural distortions in enhancement of transition temperature in FeSe_1−*x*_Te_*x*_ superconductors. Supercond. Sci. Technol.

[20] Zhong H-X, Gao S, Shi J-J, Yang L (2015). Quasiparticle band gaps, excitonic effects, and anisotropic optical properties of the monolayer distorted 1 T diamond-chain structures ReS_2_ and ReSe_2_. Phys. Rev. B.

[21] Deng K (2016). Experimental observation of topological Fermi arcs in type-II Weyl semimetal MoTe_2_. Nat. Phys.

[22] Bahramy MS (2018). Ubiquitous formation of bulk Dirac cones and topological surface states from a single orbital manifold in transition-metal dichalcogenides. Nat. Mater..

[23] Rï£·egg A, Mitra C, Demkov AA, Fiete GA (2013). Lattice distortion effects on topological phases in (LaNiO_3_)_2_/(LaAlO_3_)_*N*_ heterostructures grown along the [111] direction. Phys. Rev. B.

[24] Kargarian M, Wen J, Fiete GA (2011). Competing exotic topological insulator phases in transition-metal oxides on the pyrochlore lattice with distortion. Phys. Rev. B.

[25] Yang B-J, Kim YB (2010). Topological insulators and metal-insulator transition in the pyrochlore iridates. Phys. Rev. B.

[26] Chen, P. *et al.* Charge density wave transition in single-layer titanium diselenide. *Nat Commun.* **6**, 8943 (2015) 10.1038/ncomms9943 PMC4660365 26568512

[27] Gu Y, Katsura Y, Yoshino T, Takagi H, Taniguchi K (2015). Rechargeable magnesium-ion battery based on a TiSe_2_-cathode with d-p orbital hybridized electronic structure. Sci. Rep.

[28] Sun X, Bonnick P, Nazar LF (2016). Layered TiS_2_ Positive Electrode for Mg Batteries. ACS Energy Lett.

[29] Lv R (2015). Transition Metal Dichalcogenides and Beyond: Synthesis, Properties, and Applications of Single- and Few-Layer Nanosheets. Acc. Chem. Res..

[30] Whittingham, M. S. Chalcogenide battery. *US Patent**No*. 4,009,052 (1977).

[31] Chianelli RR, Scanlon JC, Thompson AH (1975). Structure refinement of stoichiometric TiS_2_. Mater. Res. Bull..

[32] Arnaud Y, Chevreton M (1981). Etude comparative des composï£·s TiX_2_ (X = S, Se, Te). Structures de TiTe_2_ et TiSeTe. J. Solid State Chem..

[33] Ishioka J (2010). Chiral Charge-Density Waves. Phys. Rev. Lett..

[34] Castellan J-P (2013). Chiral Phase Transition in Charge Ordered 1T-TiSe_2_. Phys. Rev. Lett..

[35] Cercellier H (2007). Evidence for an Excitonic Insulator Phase in 1T-TiSe_2_. Phys. Rev. Lett..

[36] Wilson JA (1978). Modelling the contrasting semimetallic characters of TiS_2_ and TiSe_2_. Physica status solidi (b).

[37] Wilson JA (1977). Concerning the semimetallic characters of TiS_2_ and TiSe_2_. Solid State Commun..

[38] Hughes HP (1977). Structural distortion in TiSe_2_ and related materials-a possible Jahn-Teller effect?. J. Phys. C: Solid State Physics.

[39] Rossnagel K, Kipp L, Skibowski M (2002). Charge-density-wave phase transition in 1T-TiSe_2_ Excitonic insulator versus band-type Jahn-Teller mechanism. Phys. Rev. B.

[40] Bianco R, Calandra M, Mauri F (2015). Electronic and vibrational properties of TiSe_2_ in the charge-density-wave phase from first principles. Phys. Rev. B.

[41] Rohwer T (2011). Collapse of long-range charge order tracked by time-resolved photoemission at high momenta. Nature.

[42] Wu ZY, Ouvrard G, Moreau P, Natoli CR (1997). Interpretation of preedge features in the Ti and S K-edge x-ray-absorption near-edge spectra in the layered disulfides TiS_2_ and TaS_2_. Phys. Rev. B.

[43] Wan C (2015). Flexible n-type thermoelectric materials by organic intercalation of layered transition metal dichalcogenide TiS_2_. Nat. Mater..

[44] Bullett DW (1978). Electronic band structure and bonding in transition metal layered dichalcogenides by atomic orbital methods. J. Phys. C: Solid State Phys..

[45] Shkvarin AS (2012). Electronic structure of titanium dichalcogenides TiX_2_ (X = S, Se, Te). Journal of Expt. and Theo. Phys.

[46] Stavitski E, de Groot FMF (2010). The CTM4XAS program for EELS and XAS spectral shape analysis of transition metal L edges. Micron.

[47] Wilson JA, Yoffe AD (1969). The transition metal dichalcogenides discussion and interpretation of the observed optical, electrical and structural properties. Adv. Phys..

[48] Khomskii, D. I. *Transition Metal Compounds* (Cambridge University Press).

[49] Simunek A, Sipr O, Bocharov S, Heumann D, Drager G (1997). Unoccupied electron states of TiS_2_ studied by means of polarized x-ray absorption. Phys. Rev. B.

[50] Clerc DG, Poshusta RD (1996). Periodic Hartree-Fock Study of TiS_2_. J. Phys. Chem..

[51] Fang CM, de Groot RA, Haas C (1997). Bulk and surface electronic structure of 1T-TiS_2_ and 1T-TiSe_2_. Phys. Rev. B.

