# Supplementary material for: Anomalous orbital structure in two-dimensional titanium dichalcogenides
Source: Sci Rep. 2019 Feb 13;9:1896. doi: 10.1038/s41598-018-37248-5 (PMC6374443; doi:10.1038/s41598-018-37248-5)
Supplement: Supplementary file 1 — Supporting information [file 41598_2018_37248_MOESM1_ESM.pdf]

Supporting Information for
Anomalous orbital structure in titanium di-chalcogenides

Banabir Pal,[1,*] Yanwei Cao,[1,2,†] Xiaoran Liu,[1] Fangdi Wen,[1] M. Kareev,[1] A. T. N'Diaye,[3] P. Shafer,[3] E. Arenholz,[3] and J. Chakhalian[1]

[1]*Department of Physics and Astronomy, Rutgers University, Piscataway, New Jersey 08854, USA*

[2]*Ningbo Institute of Materials Technology and Engineering, Chinese Academy of Sciences, Ningbo, Zhejiang 315201, China*

[3]*Advanced Light Source, Lawrence Berkley National Laboratory, Berkeley, California 94720, USA*

(Dated: July 3, 2018)

X-RAY PHOTOELECTRON SPECTROSCOPY ON DIFFERENT CORE LEVEL:

In order to exclude the presence of anion vacancies possibly detrimental for materials properties, X-ray photoelectron spectroscopy (XPS) measurements were carried out on three representative TiX_2 systems. Fig. S1 shows S $2p$, Se $3d$ and Te $3d$ core level spectra for TiS_2, $TiSe_2$, and $TiTe_2$ systems, respectively. Like the Ti $2p$ core level spectra shown in the main manuscript, the doublet features in each spectrum of Fig. S1 can also be described by two Gaussian-Lorentz line profile. Within the accuracy of the experiment, the presence of single charge state anions in these three systems excludes the presence of chalocogen vacancies in our systems.

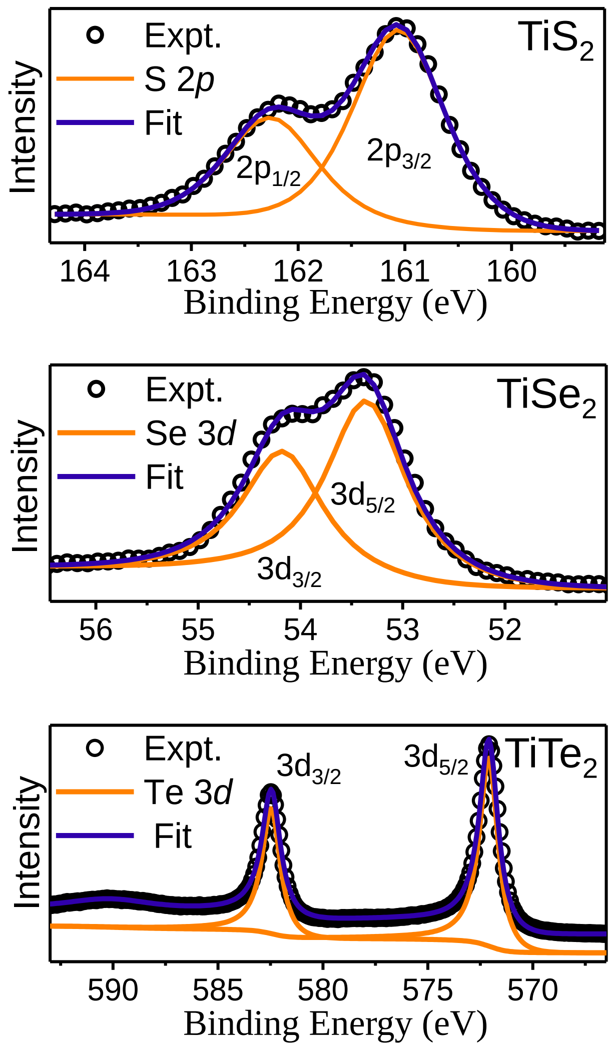

FIG. S1. X-ray photoelectron spectra of S $2p$, Se $3d$ and Te $3d$ core level. Each spectrum is decomposed with the Lorentzian function convoluted with Gaussian functions.

ESTIMATION OF THE SUB-BAND SPLITTING:

The extent of the sub-band splitting estimated from the polarization dependent XAS measurements is shown schematically in Fig. S2.

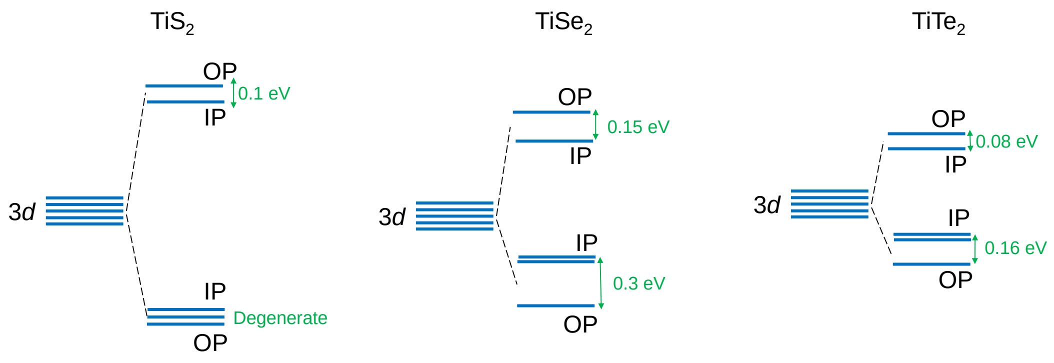

FIG. S2. Schematic representation of $3d$ band in the atomic limit.

It is important to note that this calculation of the sub-band splitting has been deduced from the L_3 edge XAS spectra. Surprisingly, the magnitude of the sub-band splitting shows a non monotonic behavior in these systems. Specifically, though $TiTe_2$ has the largest trigonal distortion, the magnitude of the sub-band splitting is highest in case of $TiSe_2$.

ATOMIC MULTIPLET CLUSTER CALCULATION:

As alluded in the main manuscript, atomic multiplet cluster calculations were carried out using the charge transfer multiplet program (CTM4XAS) software [1] to simulate the experimental XAS spectra. A TiX_6 cluster with O_h symmetry was used for this purpose. Both experimental and calculated spectra are shown in Fig. 2(b) in the main manuscript. The relevant atomic parameter that were obtained after fitting each experimental spectrum are summarized in Table S1 for all three systems. The term 10Dq represents the crystal field splitting strength of the TiX_6 octahedra. In a typical octahedral environment 10Dq is defined

TABLE S1. Best parameters obtained after multiplet cluster calculations of Ti^{4+} in octahedral symmetry.

**System**	10Dq	U_{dd}-U_{pd}	F_{dd}, F_{pd}, G_{pd}
TiS_2	2.2	-1.35	0.8, 0.8, 0.8
$TiSe_2$	2.0	-2.15	0.8, 0.8, 0.8
$TiTe_2$	1.75	-3.35	0.8, 0.8, 0.8

as the energy difference between the lower energy t_{2g} states and the higher energy e_g states. The parameter U_{dd} represents the on-site coulomb interaction energy and is expressed with Hubbard U; in addition, U_{pd} defines the core hole interaction potential. XAS calculation, For cluster calculations the relevant parameter is the energy difference (U_{pd}-U_{dd}). Reduction (20%) in Slater integral was controlled by using F_{dd}, F_{pd}, G_{pd}. Here, F_{dd} and F_{pd}, represent direct coulomb Slater integrals whereas G_{pd} represents the exchange coulomb Slater integral.

* bp435@physics.rutgers.edu
† ywcao@nimte.ac.cn

[1] E. Stavitski and F. M. F. de Groot, Micron, **41**, 687-694. (2010)
